# Non-Invasive Prediction of Choledocholithiasis Using 1D Convolutional Neural Networks and Clinical Data

**DOI:** 10.3390/diagnostics14121278

**Published:** 2024-06-17

**Authors:** Enrique Mena-Camilo, Sebastián Salazar-Colores, Marco Antonio Aceves-Fernández, Edgard Efrén Lozada-Hernández, Juan Manuel Ramos-Arreguín

**Affiliations:** 1 Facultad de Ingeniería, Universidad Autónoma de Querétaro, Querétaro 76010, Mexico; emena05@alumnos.uaq.mx (E.M.-C.); marco.aceves@uaq.mx (M.A.A.-F.); jramos@mecamex.net (J.M.R.-A.); 2 Centro de Investigaciones en Óptica, León 37150, Mexico; 3 Hospital Regional de Alta Especialidad del Bajío, León 37660, Mexico; edgardlozada2@gmail.com

**Keywords:** choledocholithiasis, convolutional neural network, endoscopic retrograde cholangiopancreatography, risk prediction

## Abstract

This paper introduces a novel one-dimensional convolutional neural network that utilizes clinical data to accurately detect choledocholithiasis, where gallstones obstruct the common bile duct. Swift and precise detection of this condition is critical to preventing severe complications, such as biliary colic, jaundice, and pancreatitis. This cutting-edge model was rigorously compared with other machine learning methods commonly used in similar problems, such as logistic regression, linear discriminant analysis, and a state-of-the-art random forest, using a dataset derived from endoscopic retrograde cholangiopancreatography scans performed at Olive View–University of California, Los Angeles Medical Center. The one-dimensional convolutional neural network model demonstrated exceptional performance, achieving 90.77% accuracy and 92.86% specificity, with an area under the curve of 0.9270. While the paper acknowledges potential areas for improvement, it emphasizes the effectiveness of the one-dimensional convolutional neural network architecture. The results suggest that this one-dimensional convolutional neural network approach could serve as a plausible alternative to endoscopic retrograde cholangiopancreatography, considering its disadvantages, such as the need for specialized equipment and skilled personnel and the risk of postoperative complications. The potential of the one-dimensional convolutional neural network model to significantly advance the clinical diagnosis of this gallstone-related condition is notable, offering a less invasive, potentially safer, and more accessible alternative.

## 1. Introduction

Choledocholithiasis is characterized by the obstruction of the common bile duct, which can be either partial or complete, owing to the presence of gallstones. Often emerging as a complication of cholelithiasis—gallstone formation in the gallbladder—this condition represents a significant health challenge. It is estimated that between 10% and 20% of gallstone cases will present common bile duct stones (CBDSs) [1,2]. Various studies conducted worldwide have determined that the prevalence of this condition ranges from 9% to 21%, varying by region of study [3,4,5].

At present, endoscopic retrograde cholangiopancreatography (ERCP) stands as the diagnostic method with the highest accuracy in identifying choledocholithiasis. Various studies support that ERCP has a sensitivity that fluctuates between 80% and 93%, and it boasts a specificity reaching 100% [2,6,7]. While the efficacy of ERCP is high, the procedure is not without its challenges. These include the requirement for both specialized equipment and highly trained personnel. Moreover, there are risks of postoperative complications associated with ERCP. Such complications may range from pancreatitis and perforations of the duodenum to internal bleeding. In the most severe cases, these complications can have fatal outcomes [6,7,8,9,10]. Despite these risks, ERCP remains a critical tool in the management and diagnosis of biliary tract diseases, underscoring the importance of skilled operation and patient selection.

Although choledocholithiasis typically arises as a complication of cholelithiasis, predictors of the former are often derived from those for the latter [11,12,13,14]. However, the validity of these extrapolated predictors has not been adequately assessed in the context of choledocholithiasis. Furthermore, considering that only a minority fraction of patients with cholelithiasis will develop choledocholithiasis, it is crucial to have non-invasive and specific prediction methods at our disposal [6,15].

Well-known institutions like the American Society for Gastrointestinal Endoscopy (ASGE) have suggested criteria based on clinical indicators to categorize the suspicion levels for choledocholithiasis. These criteria consider various factors, including the presence of stones in the common bile duct (CBD), the occurrence of cholangitis, total bilirubin levels, CBD dilation, pancreatitis, and the patient’s age. Using these factors, the risk of choledocholithiasis is classified into low, medium, and high categories. Although these criteria have been assessed in various studies with a sensitivity of up to 90.9% and a specificity of up to 26.7% [16,17,18], they are regarded as an effective and non-invasive option for diagnosing choledocholithiasis, albeit not at the level of the ERCP method. In this context, machine learning emerges as a powerful ally, offering significant contributions to the medical field by leveraging its pattern recognition strengths to analyze clinical data.

The convergence of clinical expertise and machine learning innovation has ushered in a new era of groundbreaking advancements in medical diagnostics. This is exemplified by G. Hernández-Nava et al. [19], who developed a novel and highly effective technique for detecting epileptic seizures using EEG signals. S. Leyva-López et al. [20], on the other hand, employed deep learning segmentation techniques to successfully predict the extent of damage caused by idiopathic pulmonary fibrosis. Additionally, M. Fraiwan et al. [21] leveraged clinical data to design a high-performance dual classifier capable of identifying patients requiring abdominal surgery and the associated risk level.

In connection with the study topic of this paper, various studies of significance have been identified. P. Jovanovic et al. [22] proposed a multilayer perceptron with nine inputs. This model reached a sensitivity of 92.74% and a specificity of 68.42%. M. Vukicevic et al. [23] developed an artificial neural network using various clinical parameters as inputs. Their approach achieved a sensitivity of 88.20% and a specificity of 95.80%. V. S. Akshintala et al. [24] implemented a random forest model based on a gradient boosting machine and XGBoost, incorporating 12 clinical parameters as inputs. The model attained a sensitivity of 82.40% and a specificity of 63.30%. Dalai et al. [16] performed a comparison of the performance of several machine learning models. Their top model was a random forest that used eight clinical parameters as inputs, achieving a sensitivity of 77.70% and a specificity of 75.00%.

Designing classifiers based on clinical data is crucial. They provide an accessible, non-invasive diagnostic method for all, which not only significantly enhances patient quality of life but also diminishes the risks associated with complications from ERCP. Therefore, the aim of this study is to introduce a machine learning algorithm designed to determine a patient’s risk of developing choledocholithiasis based on clinical parameters. This goal is achieved using one-dimensional convolutional neural networks (1D CNNs), with the aim of outperforming existing machine learning methods such as logistic regression, linear discriminant analysis, and random forest, as well as surpassing the current best human-established criteria set by the ASGE.

This paper is organized as follows: Section 2 provides the background. The materials and methods used are detailed in Section 3. The results are laid out in Section 4. A discussion of these results is found in Section 5. Finally, the conclusions are drawn in Section 6.

## 2. Background

### 2.1. Logistic Regression

Logistic regression (LR) is a regression analysis method used to predict the outcome of a categorical variable based on independent variables. It employs a logistic function to convert the linear combination of the independent variables into a probability, which consistently ranges between 0 and 1. This logistic function is defined in Equation (1) [25,26]:

(1) $$y=\frac{1}{1+e^{-(\beta_{0}+\beta_{1}x_{1}+\beta_{2}x_{2}+...+\beta_{n}x_{n})}}$$

where *y* is the probability of the event occurring; $\beta_{0},\ldots ,\beta_{n}$ are the model coefficients; and $x_{1},\ldots ,x_{n}$ are the independent variables [25,26].

The model coefficients are estimated using methods such as the maximum likelihood estimation. These coefficients indicate the strength and direction of the relationship between the independent variables and the dependent variable.

The output of logistic regression is the probability of the response being 1. Consequently, a decision threshold must be defined for classification. This threshold is commonly set at 0.5 for binary classifications [25,26].

Among the advantages of LR, we find that it is a relatively simple model to understand and apply; it is a versatile model usable in a broad range of applications; and it is a robust model that is relatively insensitive to missing data or the data distribution.

LR has a wide range of applications, extending into the medical field, where it has demonstrated excellent performance in classification procedures and feature extraction, as evidenced in the works of [27,28].

### 2.2. Linear Discriminant Analysis

Linear discriminant analysis (LDA) is a machine learning method designed to classify observations by assuming a similar distribution within each class [29,30].

In general terms, LDA operates in four steps:

1. Observations from each class are assumed to follow a normal distribution.
2. The mean and covariance matrix for each class are calculated.
3. Linear discriminant functions are computed to optimally separate the classes.
4. The classification of new observations is performed using these discriminant functions.

The linear discriminant functions conform to Equation (2):

(2) $$f_{k}\left(x\right)=w_{k}^{T}x-b_{k}$$

where $f_{k}\left(x\right)$ is the discriminant function for class *k*; $w_{k}$ is the weight vector for class *k*, calculated using the covariance matrix; and $b_{k}$ is the bias term for class *k*, computed using the class mean [29,30].

Predictions are made by assigning an observation to class *k* for which the discriminant function is highest [29,30].

LDA’s advantages include its simplicity, computational efficiency, and high accuracy when class observations have distinct distributions. It has been effectively applied in medical fields, such as in complex tasks like cancer classification [31,32], and for enhancing other predictive algorithms [33].

### 2.3. Convolutional Neural Network

A convolutional neural network (CNN) is an artificial neural network specialized in processing structured data like grids, applicable to time series, images, or videos. It is named “convolutional” due to its use of convolution operations, which involves sliding a filter across the grid dimensions to extract local features [34,35,36].

In particular, 1D CNNs are designed to process one-dimensional data, such as sequences, signals, and text. In these networks, the convolution operation is applied along the temporal dimension of the data [34,35,36].

While 1D CNNs have shown significant utility in temporal series applications, their effectiveness extends to structured tabular data, including medical data for feature extraction using various 1D CNN filters [37,38,39,40]. The effectiveness of the analysis of structured tabular data is based on the convolution operation. This operation seeks to identify patterns not only in the target variables but also in the characteristics being analyzed. This allows the patterns present in the variables used to be exploited as useful information in the classification process.

CNNs are notable for their data generalization capabilities, suitability for supervised learning, and robustness against noise and interference, making them highly effective in real-world scenarios.

## 3. Materials and Methods

In this section, we outline our methodology, centering on the analysis of a detailed dataset from Olive View–University of California, Los Angeles (UCLA) Medical Center, and the rigorous development and validation of various 1D CNN architectures. This approach ensures a comprehensive and precise evaluation of our machine learning models in classifying choledocholithiasis.

### 3.1. Study Population

The dataset used for this study was voluntarily provided by the Olive View–UCLA Medical Center research group. This dataset gathers information from all ERCPs performed from 1 November 2015 to 31 December 2019 on adult patients [16]. It comprises 550 instances, 26 attributes, and a decision variable. The decision variable is a value indicating whether a patient has choledocholithiasis confirmed by ERCP (confirmation is defined as the presence of stones, debris, or sludge in the common bile duct). The attributes include the following:

- Demographic data: age, gender, and race.
- Clinical variables: body mass index, diabetes mellitus, cirrhosis, peak bilirubin, the presence of gallstones in non-invasive imaging tests, the diameter of the common bile duct, and the existence of the gallbladder.
- Annotations/comments: mixed texts made by specialists in the field.

### 3.2. Data Preprocessing

An initial exploratory analysis of the dataset was conducted. It was determined that attributes related to annotations and comments were irrelevant to the study. Therefore, the following attributes were removed: id, the date of the surgical procedure, reasons for the surgical intervention, and the specialist’s comments. Null information in the target variable was also identified and removed.

During the exploration, the presence of non-standard notations was identified. For example, values such as "x", "0x", and similar variants were treated as null data. Additionally, categorical data in text format were converted into integer values.

Subsequently, it was found that six attributes had more than 25% missing data, leading to the decision to eliminate them. For the remaining attributes, traditional imputation methods were employed: categorical attributes were imputed using the mode, and continuous attributes were imputed using the mean.

With a complete dataset in hand, normalization techniques were applied to the continuous attributes. The z-score method was chosen for this purpose, as it standardizes the continuous data to have a final mean of 0 and a standard deviation of 1.

The resulting dataset from preprocessing consists of 292 instances, with 12 attributes and a decision variable. The distribution of this decision variable indicates 251 positive cases (86%) and 41 negative cases (14%), evidencing a remarkable class imbalance. This imbalance poses a significant challenge, whose possible solutions will be addressed in later sections.

Finally, two copies of the preprocessed dataset were created. The first copy was intended for cross-validation processes, while the second was reserved for final validation processes and model export.

For cross-validation, we divided the first copy into 10 folds using the stratified k-fold technique. This methodology implies that 9 folds are used to train the model, while the remaining fold is reserved as a test set. Once training with the training set was completed, we evaluated the model with the test set and temporarily stored the results. Then, we swapped the test model with one of the training folds that was not previously used as a test set, reset the model weights, and retrained. At the end of training, we evaluated the model with the test set and stored these results. This procedure was repeated until all folds had been used as test sets. Then, we averaged the results of each experiment to obtain the average performance of our model. In addition, we repeated this cross-validation process 100 times to allow the k-fold mechanism to select different elements in each run, which gives us a more accurate estimate of the model performance.

On the other hand, we divided the second copy of our data into two segments: 80% for training and 20% for testing. This set was used to train an untrained copy of the final model. During this training process, we obtained graphs showing the evolution of the cost in the training and validation sets. Once training was completed, we evaluated the model using the test data (data not observed by the model), which involved generating the ROC curve of the final model.

### 3.3. Design of Network Architectures

Different CNN architectures were experimented with, using 1D kernels in the convolutional layers. The variations focused on architectures with between 2 and 3 internal layers and models with 32, 64, 128, and 256 filters. Despite these variations, the kernel size was kept constant, being (1, 12) for the input and (1,1) for the output. The general training parameters were fixed in all variations: 10 epochs, a batch size of 16, the Adam optimizer, a learning rate of 0.001, and the binary cross-entropy loss function. These parameters were determined through prior experimentation, employing an early stopping criterion that halted training if the validation loss did not improve by at least 0.025 within 3 consecutive epochs.

As a result of the design process described in the previous paragraph, the 1D CNN that performed best is detailed in Table 1, demonstrating a configuration of 2 internal layers. It is important to mention that the selection of this architecture was based on a meticulous process of 10-fold k-fold cross-validation that will be described in later sections.

### 3.4. Training and Validation of Architectures

To select the best CNN architecture, a 10-fold cross-validation was implemented on the complete dataset. This cross-validation process was performed following a stratified strategy, which ensures that the percentage of each class is preserved in each fold, allowing us to deal with the class mismatch present in the data.

In each fold, each model was compiled, trained, and evaluated using various metrics: accuracy, sensitivity, specificity, positive predictive value (PPV), negative predictive value (NPV), F1 score, and area under the curve (AUC). This process was replicated for all combinations of hyperparameters previously described. Upon completing the cross-validation, the mean performance of the metrics for each architecture was determined, and the one that showed the greatest consistency in results across folds was selected.

In parallel, two conventional machine learning models were trained: LR and LDA. Both models underwent the same cross-validation protocol used for the CNN architectures.

Once the optimal architecture was defined, it was re-evaluated using the test set, which represented 20% of the original data segregated during preprocessing. This final evaluation aimed to determine the area under the ROC curve for the 1D CNN, LR, and LDA architectures.

Additionally, a graph was generated to contrast the performance of these models with the results of relevant studies in the field.

## 4. Results

### 4.1. Analysis of Study Population

Regarding the data preprocessing performed, it is noted that the characteristics, once normalized, operate within comparable ranges, and thanks to imputation, data gaps have been mitigated. These interventions have paved the way for the effective training of machine learning algorithms, optimizing their performance.

In total, the dataset consists of 292 unique records, with a distribution of 251 cases (86%) confirmed with choledocholithiasis and 41 cases (14%) without evidence of it. The detailed breakdown of the attributes, segmented by study category, is presented in Table 2. This table specifies the units for each attribute, as well as the median and interquartile range for those attributes of a continuous nature. For categorical attributes, the table shows the frequency and the corresponding percentage for each category.

In addition, during the analysis of the study data, the importance of each of the attributes in the classification process was determined. For this purpose, Pearson’s and Kendall’s correlations were used as importance metrics. Table 3 shows the importance of each characteristic, showing the characteristic with the highest contribution in first place and the characteristic with the lowest contribution in last place.

Examining the data presented in Table 3, it is evident that the intraductal filling variable exhibits the most prominent correlation with our variable of interest in terms of both Pearson’s correlation coefficient (assessing linear relationship) and Kendall’s correlation coefficient (assessing rank correlation). Furthermore, it is observed that both gallbladder and common bile duct diameters (assessed by ultrasound and ERCP) show significant correlations according to both calculated indices. These findings provide us with insight into the patterns considered by the architectures proposed in this study when generating their predictive results.

### 4.2. LR and LDA Models

The LR and LDA models were trained in parallel with the CNN architectures. For LR, the resulting equation after training is established in Equations (3) and (4):

(3) $$\begin{array}{c} y=\frac{1}{1+e^{-t}} \end{array}$$

(4) $$\begin{array}{cc} t= & -1.7832+0.0160x_{1}-0.2718x_{2}+0.2439x_{3}+0.1170x_{4}\\ \; & -0.6438x_{5}-0.1492x_{6}+0.6289x_{7}-0.0755x_{8}+0.0217x_{9}\\ \; & +0.2316x_{10}+2.5761x_{11}+0.1447x_{12} \end{array}$$

Meanwhile, LDA resulted in a single linear combination of various features, which is defined by Equation (5):

(5) $$\begin{array}{cc} f(x)= & -3.5391+0.1828x_{1}-0.8358x_{2}+0.3553x_{3}+0.1245x_{4}-1.3415x_{5}-1.2359x_{6}\\ \; & +0.7679x_{7}+0.2481x_{8}+0.1693x_{9}+0.0525x_{10}+4.0062x_{11}+0.6648x_{12} \end{array}$$

### 4.3. One-Dimensional CNN Model

**Figure 1:**
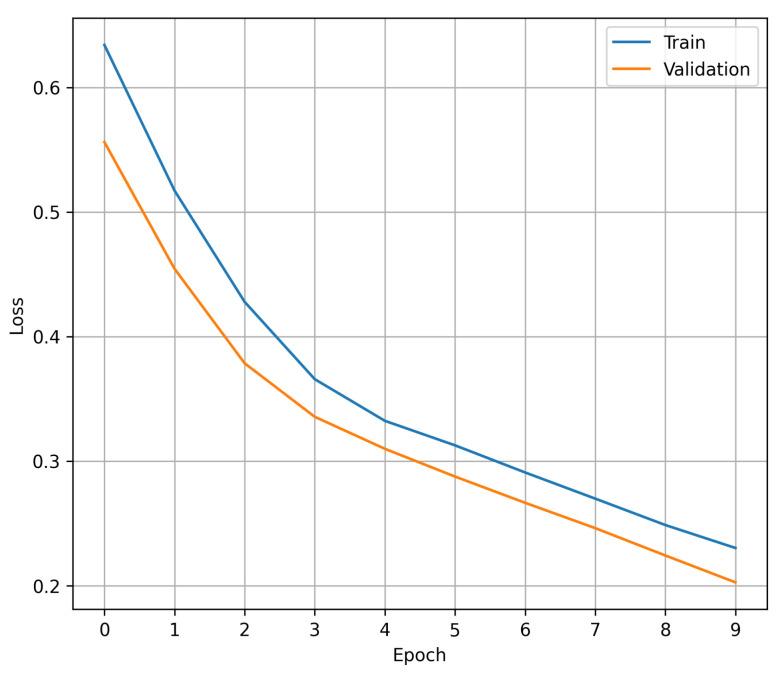
The loss during the training process of the 1D CNN model. In blue, loss on the training set. In orange, loss in the validation set.

Figure 1 shows the process of convergence of the loss during each of the trained epochs: in blue, the value of the loss in the training set is shown, while orange represents the value of the loss in the validation set. The data shown in the graph demonstrate that the early stopping criterion experimented with prior to the final training prevents the proposed 1D CNN from showing signs of overfitting, with 10 epochs being an adequate number to maintain a balance between achieving the best performance and avoiding overfitting. It should be noted that the claim of avoiding over-adjustment arises from the fact that within the 10 training epochs, there is no point at which the validation cost increases while the training cost decreases.

### 4.4. Performance of Models

In order to show the feasibility of the results obtained, 100 trials were carried out where the 10-fold cross-validation process was replicated for each of the architectures proposed in this study (1D CNN, LDA, and LR). For each of the trial processes, the mean values of each of the metrics were obtained, obtaining the mean performance of each architecture in each of the trials. Finally, the mean performance of each trial was averaged with the rest, providing the mean performance of each architecture after the 100 trials. It is worth mentioning that the metrics used to obtain the mean values were derived from the validation sets of each of the k-fold experiments.

Table 4 compares the performance of the proposed architecture with traditional machine learning models and results reported in studies using the same database. It integrates the findings of [16] for their standout machine learning model, along with evaluations based on ASGE criteria. These results show the mean value obtained at the end of the 100 experiments, as well as their standard deviation (std).

**Figure 2:**
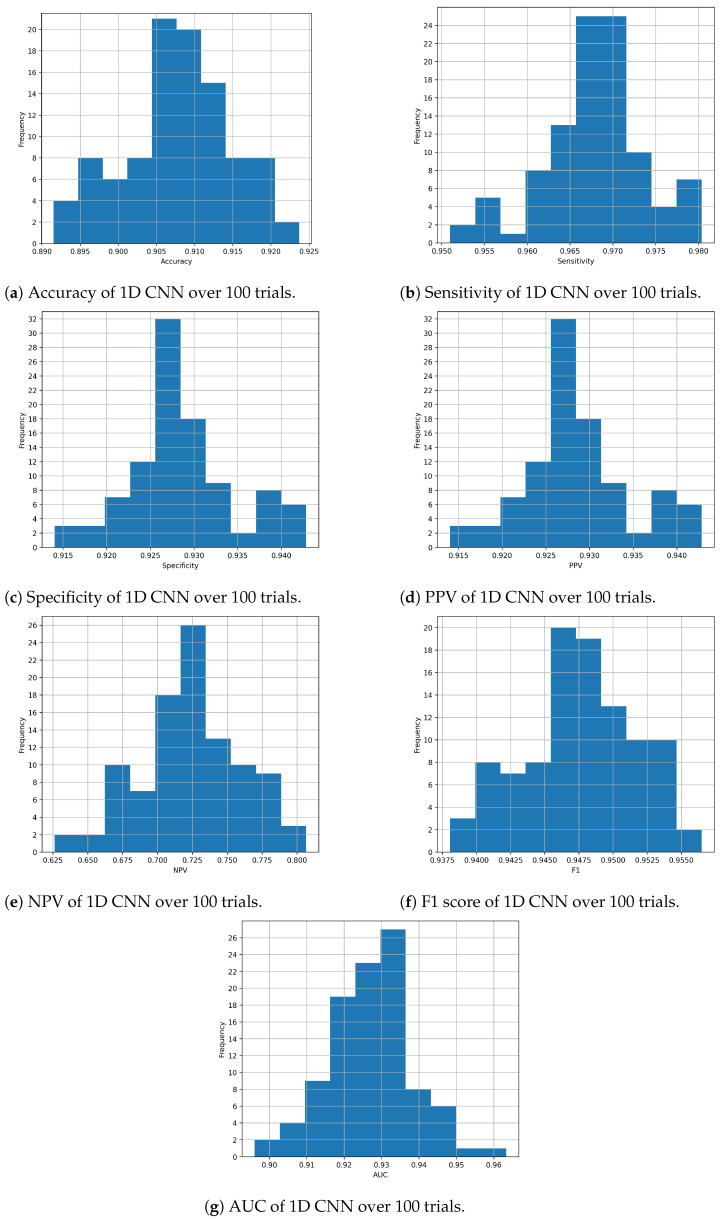
Performance of 1D CNN over 100 trials. (**a**) Sensitivity. (**b**) Accuracy. (**c**) Specificity. (**d**) PPV. (**e**) NPV. (**f**) F1 score. (**g**) AUC.

Considering the proposed 1D CNN architecture in particular, Figure 2a–g show the distribution of each metric during the evaluation process with the 100 experiments. These histograms allow us to observe how the performance of the architecture varies, demonstrating that the results obtained are not random.

**Figure 3:**
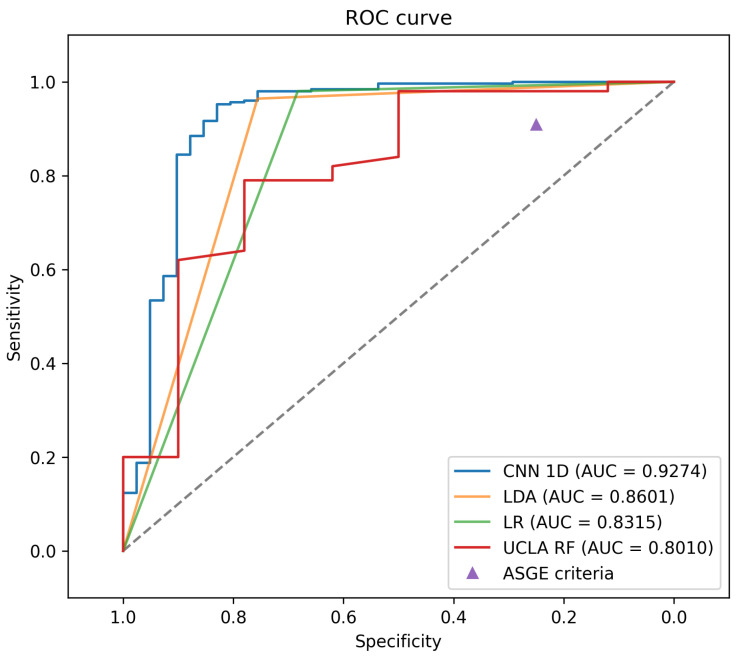
ROC curves of the models trained for predicting choledocholithiasis. The ASGE criteria are represented as a single point because this information comes from relevant studies.

Additionally, Figure 3 illustrates the ROC curves of the models mentioned in Table 4. These ROC curves are the result of having evaluated each of the models in the test set and include the value of the area under the curve (AUC) of each model.

## 5. Discussion

Reviewing the performance comparison in Table 4, the effectiveness of the proposed architecture in classifying choledocholithiasis is apparent.

In terms of accuracy, the 1D CNN shows an average value of 90.77%, indicating that, overall, it correctly classifies 90.77% of the test samples. This figure is competitive with LR and LDA, although the latter two show a slightly higher accuracy, with values of 92.22% and 92.53%, respectively.

In terms of sensitivity, which measures the ability of the model to correctly identify positive samples, the 1D CNN exhibits outstanding performance, with an average value of 96.77%. This result indicates that the CNN has a high ability to detect positive samples within the dataset, slightly outperforming LR and LDA in this metric.

In terms of specificity, which evaluates the ability of the model to correctly identify negative samples, the 1D CNN shows a similarly strong performance, with an average value of 92.86%.

Regarding PPV and NPV, which measure the proportion of positive and negative predictions that are correct, respectively, the 1D CNN shows average values of 92.86% and 72.35%, respectively. These figures, in conjunction with the sensitivity and specificity values, indicate that the model has high accuracy in predicting positive cases but may have more difficulty in correctly ruling out negative cases compared to LR and LDA.

In terms of the F1 score, which combines precision and sensitivity into a single metric, the 1D CNN shows an average value of 94.77%, indicating a balance between precision and the ability to correctly identify positive samples. This result highlights the effectiveness of the model in accurately classifying both classes, although it is important to note that LR and LDA also show competitive F1 scores.

As for the AUC, which evaluates the discrimination ability of the model at different classification thresholds, the 1D CNN shows an average value of 0.9270. This result indicates that the model has a high ability to distinguish between positive and negative classes, suggesting its effectiveness in binary classification.

It is important to note that the ASGE criteria set, despite not having an AUC value and, therefore, lacking a continuous ROC curve, shows a point on the graph that evidences its inferiority in specificity compared to other models, although it maintains high sensitivity.

The findings reveal that the suggested 1D CNN approach outperforms conventional machine learning methodologies such as LDA and LR, primarily due to its ability to achieve significantly higher AUC values. While it is acknowledged that, in certain metrics, alternative methods may occasionally yield superior results, a thorough examination of the outcomes derived from cross-validation underscores the notable consistency achieved by the proposed approach across diverse segments. This consistency is highlighted by the consistently lower standard deviation values exhibited by the 1D CNN across all metrics, thus affirming its heightened reliability as a predictive model.

## 6. Conclusions

In this study, we introduce a pioneering 1D convolutional neural network (CNN) tailored to leverage clinical data for the precise detection of choledocholithiasis. This pathological state, characterized by the obstruction of the common bile duct due to gallstones, presents substantial health hazards, underscoring the critical importance of swift and accurate identification to mitigate severe complications.

Our novel model distinguishes itself with exceptional proficiency in choledocholithiasis detection, as evidenced by its notable accuracy rate of 90.77% and specificity of 92.86%, accompanied by an impressive AUC value of 0.9270. These outcomes were derived from a comprehensive evaluation against alternative machine learning methodologies, utilizing a meticulously curated dataset sourced from ERCP scans conducted at Olive View–UCLA Medical Center.

The outcomes gleaned from our investigation unveil the significant potential of the 1D CNN approach in diagnosing choledocholithiasis. This triumph can be attributed to the remarkable capacity of 1D CNNs to discern inherent patterns within the input variables and effectively propagate this information through the respective internal layers. This inherent capability substantially bolsters the algorithm’s efficiency in global pattern recognition, which is further augmentable through tailored kernel configurations exhibiting high correlation values. Moreover, the innate dimensionality reduction prowess of 1D CNNs facilitates the abstraction of pivotal information, concurrently trimming down computational overheads, thus expediting the diagnostic workflow.

The ramifications of our study extend far beyond the realm of choledocholithiasis diagnosis. The non-invasive nature of the 1D CNN model heralds a safer, more accessible avenue for identifying this gallstone-related pathology, thereby poised to revolutionize prevailing clinical paradigms. This investigation not only underscores the efficacy of the 1D CNN architecture but also heralds a new era of its utilization in medical diagnostics, laying the groundwork for advancements in patient welfare and the overarching healthcare landscape.

## Figures and Tables

**Table 1:** The design of the best-performing convolutional neural network architecture.

Parameter	Value
Input (shape)	(1, 12, 1)
Internal Layer 1 (filters, kernel size)	(128, (1, 12))
Internal Layer 2 (filters, kernel size)	(512, (1, 1))
Output (shape)	(1, 1)

**Table 2:** The characteristics of all patients included in the dataset used, grouped according to ERCP findings.

Characteristic	Positive Choledocholithiasis (n = 251)	Negative Choledocholithiasis (n = 41)
Age (years), median (IQR)	46 (33–56)	47 (32–62)
Gender, n (%)		
Female	166 (66.1)	20 (48.8)
Male	85 (33.9)	21 (51.2)
Race, n (%)		
White	14 (5.6)	4 (9.8)
Hispanic/Latino	188 (74.9)	28 (68.3)
African American	4 (1.6)	0 (0)
Asian	10 (4.0)	2 (4.9)
Other	35 (13.9)	7 (17.1)
BMI, median (IQR)	29.0 (25.6–33.6)	30 (26.0–36.7)
Diabetes, n (%)		
Yes	35 (13.9)	11 (26.8)
No	216 (86.1)	30 (73.2)
Cirrhosis, n (%)		
Yes	8 (3.2)	2 (4.9)
No	243 (96.8)	39 (95.1)
Maximum bilirubin (mg/dL), median (IQR)	3.6 (1.8–5.5)	3.5 (1.9–5.8)
Biliary tract obstruction, n (%)		
Yes	135 (53.8)	19 (46.3)
No	116 (46.2)	22 (53.7)
Common bile duct diameter (mm), median (IQR)		
Ultrasound	8.2 (6–10)	6.3 (4.2–9)
ERCP	10 (9–12)	10 (8–11)
Intraductal filling, n (%)		
No	4 (1.6)	26 (63.4)
Sludge	25 (10.0)	9 (22.0)
Stone	147 (58.6)	4 (9.8)
Sludge and stone	75 (29.9)	2 (4.9)
Gallbladder, n (%)		
Yes	175 (69.7)	36 (87.8)
No	76 (30.3)	5 (12.2)

Categorical data are shown as count (n) and percentage (%) in each category, while continuous data are displayed as median and interquartile range (IQR). Abbreviations: BMI, body mass index; ERCP, endoscopic retrograde cholangiopancreatography.

**Table 3:** Importance of each characteristic in choledocholithiasis classification.

Correlation Method		Characteristic
Pearson	Kendall	
0.6300	0.4907	Intraductal filling
0.1400	0.1403	Gallbladder
0.1300	0.1136	Common bile duct diameter (Ultrasound)
0.1100	0.1075	Common bile duct diameter (ERCP)
0.0520	0.0517	Biliary tract obstruction
0.0350	0.0002	Maximum bilirubin
−0.0012	−0.0151	Age
−0.0160	0.0054	Race
−0.0320	−0.0323	Cirrhosis
−0.0590	−0.0502	BMI
−0.1200	−0.1228	Diabetes
−0.1300	−0.1253	Gender

**Table 4:** Performance of different machine learning models evaluated and the ASGE criteria.

Model	Accuracy (%)		Sensitivity (%)		Specificity (%)		PPV (%)		NPV (%)		F1 Score (%)		AUC	
	Mean	Std	Mean	Std	Mean	Std	Mean	Std	Mean	Std	Mean	Std	Mean	Std
1D CNN	90.77	0.0071	**96.77**	0.0005	**92.86**	0.0062	92.86	0.0062	72.35	0.0366	94.77	0.0040	**0.9270**	0.0112
LDA	92.22	0.0124	95.45	0.0084	77.74	0.0563	**95.45**	0.0084	77.74	0.0563	95.49	0.0074	0.8432	0.0226
LR	**92.53**	0.0110	94.29	0.0087	83.97	0.0534	94.29	0.0087	**83.97**	0.0534	**95.73**	0.0062	0.8404	0.0237
UCLA RF [16]	76.90	-	77.30	-	75.00	-	94.40	-	37.50	-	-	-	0.8010	-
ASGE [16]	80.80	-	90.90	-	25.00	-	87.00	-	33.30	-	-	-	-	-

Abbreviations: 1D CNN, one-dimensional convolutional neural network; LDA, linear discriminant analysis; LR, logistic regression; Std, standard deviation; UCLA RF, random forest designed by Olive View–UCLA Medical Center [16]; ASGE, performance of criteria from the American Society for Gastrointestinal Endoscopy, as reported by [16]. The maximum value of each of the metrics is highlighted in bold.

## Data Availability

The dataset generated for this study is available on request to the corresponding author.

## Abbreviations

The following abbreviations are used in this manuscript:

1D CNN	One-dimensional convolutional neural network
CNN	Convolutional neural network
ERCP	Endoscopic retrograde cholangiopancreatography
UCLA	University of California, Los Angeles
ROC	Receiver Operating Characteristic
AUC	Area under the curve
CBDS	Common bile duct stone
ASGE	American Society for Gastrointestinal Endoscopy
CBD	Common bile duct
LR	Logistic regression
LDA	Linear discriminant analysis
RF	Random forest
IQR	Interquartile range
BMI	Body mass index
Std	Standard deviation
